# Supplementary material for: Annexin A1 is a polarity cue that directs mitotic spindle orientation during mammalian epithelial morphogenesis
Source: Nat Commun. 2023 Jan 11;14:151. doi: 10.1038/s41467-023-35881-x (PMC9834401; doi:10.1038/s41467-023-35881-x)
Supplement: Supplementary file 1 — Supplementary Information [file 41467_2023_35881_MOESM1_ESM.pdf]

Supplementary information

Annexin A1 is a polarity cue that directs mitotic spindle orientation during mammalian epithelial morphogenesis

Maria Fankhaenel[1,2], Farahnaz S. Golestan Hashemi[1,2], Larissa Mourao[3], Emily Lucas[1,2], Manal M. Hosawi[1,2], Paul Skipp[1,2,4], Xavier Morin[5], Colinda L.G.J. Scheele[3] & Salah Elias[1,2]

Supplementary Fig. 1 Experimental design for determining the LGN interactome in mammary epithelial cells. (page 2)

Supplementary Fig. 2 ANXA1 co-distributes with S100A11, LGN, NuMA, and microtubules. (page 4)

Supplementary Fig. 3 ANXA1 acts upstream of LGN to ensure proper mitotic spindle orientation. (page 6)

Supplementary Fig. 4 Characterisation of ANXA1 expression and oriented cell divisions in human mammary epithelial cells *in situ* and 3D culture. (page 8)

Fiji custom macro for quantification of cortical protein fluorescence intensities during prometaphase. (page 10)

Representative examples of the gating strategy used to purify stable MCF-10A expressing GFP-LGN, EB3-GFP or ANXA1-mCherry (page 12)

Supplementary Fig. 1

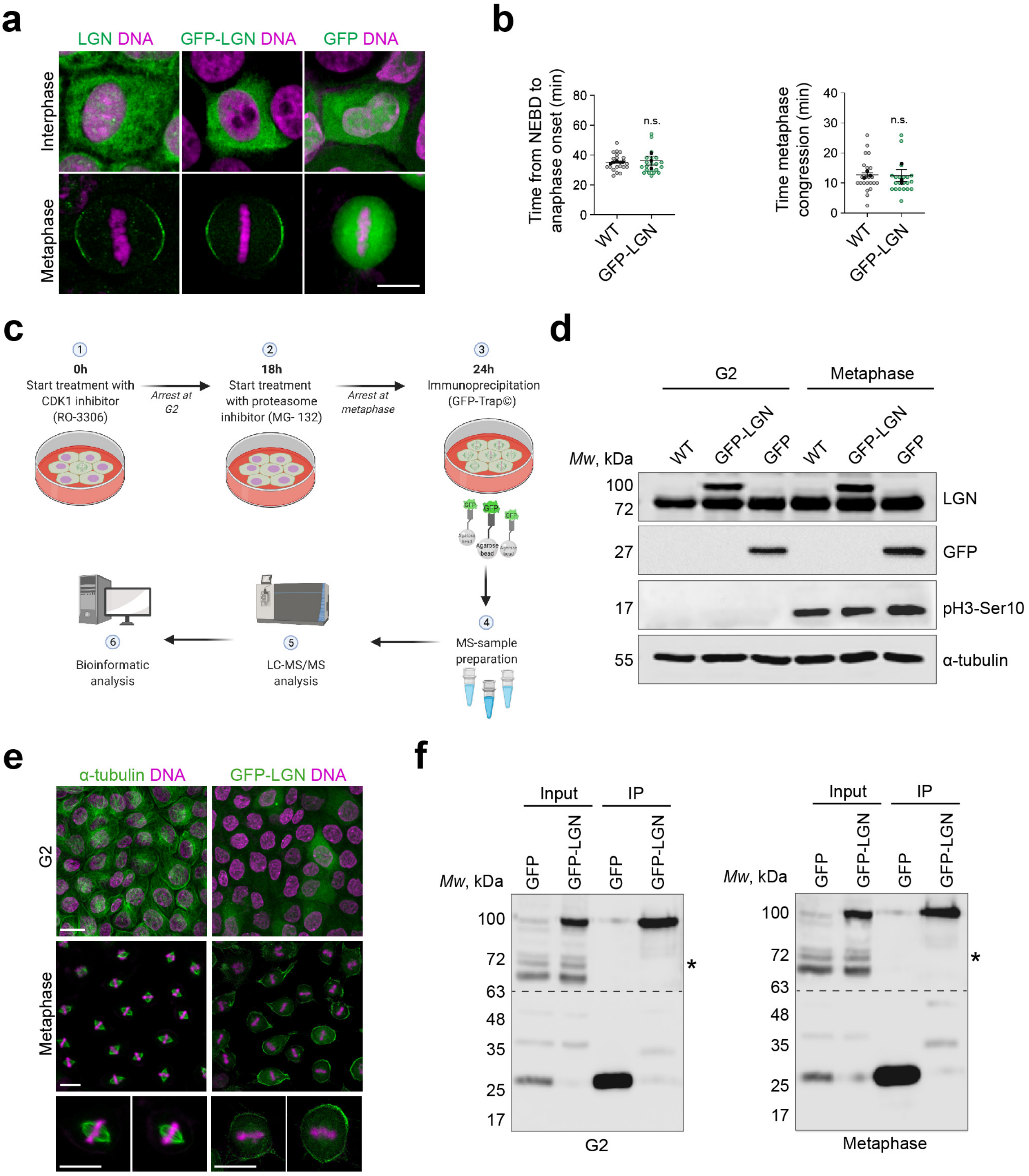

Supplementary Fig. 1 Experimental design for determining the LGN interactome in mammary epithelial cells. (a) Confocal images of representative wild type (WT) MCF-10A and the generated clonal MCF-10A stably expressing GFP-LGN or GFP in interphase and metaphase. Cells are stained for LGN or GFP (green) and counterstained with DAPI (DNA, magenta). **(b)** Time from NEBD to

anaphase (left) and duration of metaphase congression (right), determined from time-lapse microscopy in WT MCF-10A cells and clonal MCF-10A stably expressing GFP-LGN (3 independent experiments, WT: n = 23; GFP-LGN: n = 21). **(c)** Illustration showing the proteomic workflow from the synchronisation procedure to the experimental pipeline leading to LC-MS/MS and bioinformatic-based identification of the LGN interactome. Cells were treated with the CDK1 inhibitor RO-3306, yielding cells synchronised in G2 phase, then arrested in metaphase by treatment with the proteasome inhibitor MG-132. The drugs used for synchronisation were washed out prior to extract preparation for affinity purification with GFP-Trap beads. **(d)** Western blotting of extracts from WT MCF-10A and clonal MCF-10A stably expressing GFP-LGN or GFP cells synchronised in G2 phase or metaphase (3 independent experiments). **(e)** Confocal images of representative clonal MCF-10A stably expressing GFP-LGN cells synchronised in G2 phase or metaphase. Cells are stained for α-tubulin or GFP (green) and counterstained with DAPI (DNA, magenta) (3 independent experiments). **(f)** Western blotting of extracts of GFP- and GFP-LGN-bead-bound elutions from cells synchronised in G2 phase (left) or metaphase (right) (3 independent experiments). Asterisks indicate endogenous LGN in the input samples. All data are presented as mean ± s.e.m. n.s. (not significant). All scale bars, 10 µm. Source data are provided as a Source Data file.

Supplementary Fig. 2

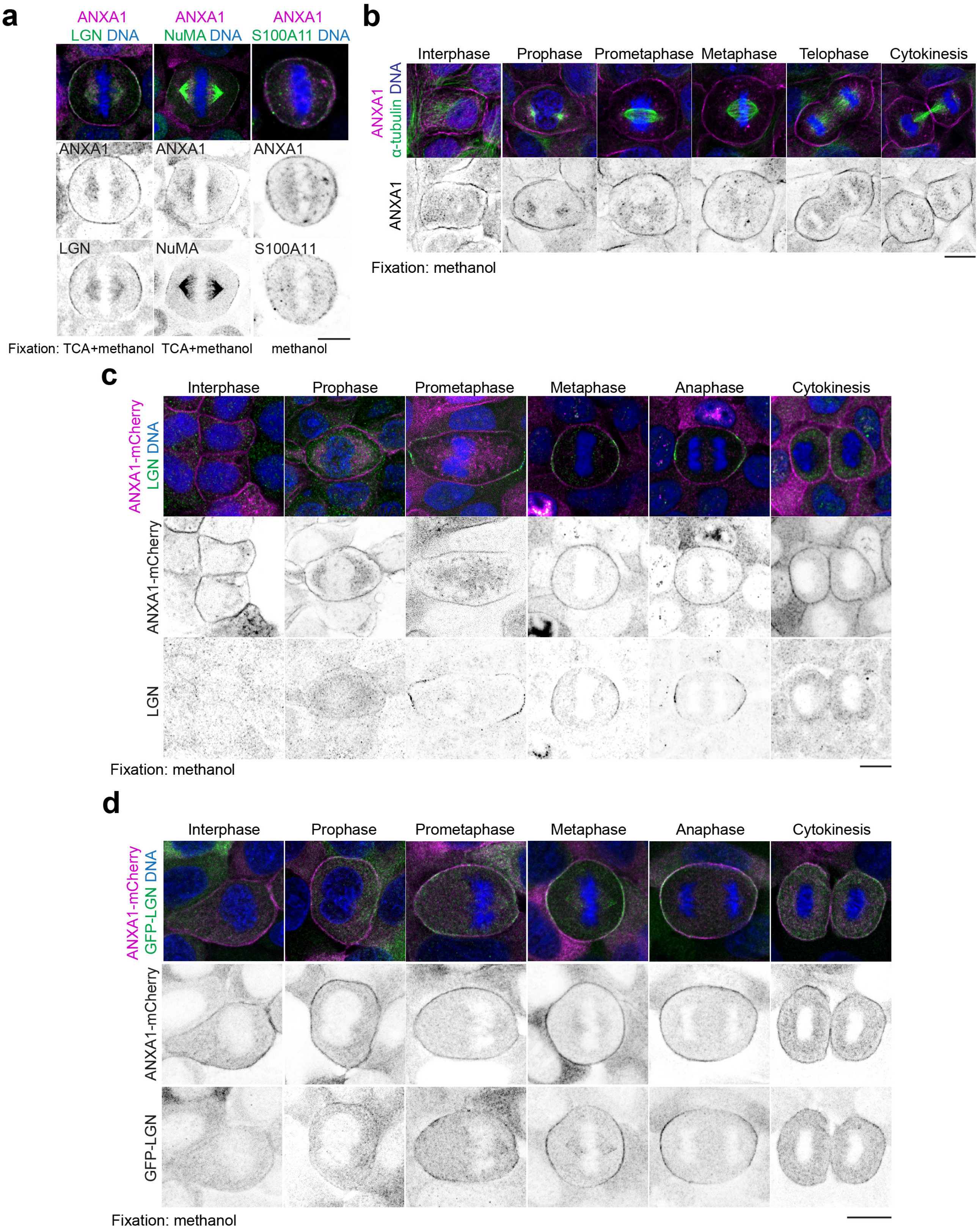

Supplementary Fig. 2 ANXA1 co-distributes with S100A11, LGN, NuMA, and microtubules. (a) Confocal images of representative MCF-10A cells stained for ANXA1 (magenta) and LGN, NuMA or

S100A11 (green), and counterstained with DAPI (DNA, blue). **(b)** Confocal images of representative MCF-10A stained for ANXA1 (magenta) and α-tubulin (green), and counterstained with DAPI (DNA, blue). **(c)** Confocal images of representative clonal MCF-10A cells stably expressing ANXA1-mCherry (magenta) stained for LGN (green) and counterstained with DAPI (DNA, blue). **(d)** Confocal images of representative clonal MCF-10A cells stably expressing ANXA1-mCherry (magenta) and GFP-LGN (green) and counterstained with DAPI (DNA, blue). All data are from 3 independent experiments. TCA (Trichloroacetic acid). All scale bar, 10 μm.

Supplementary Fig. 3

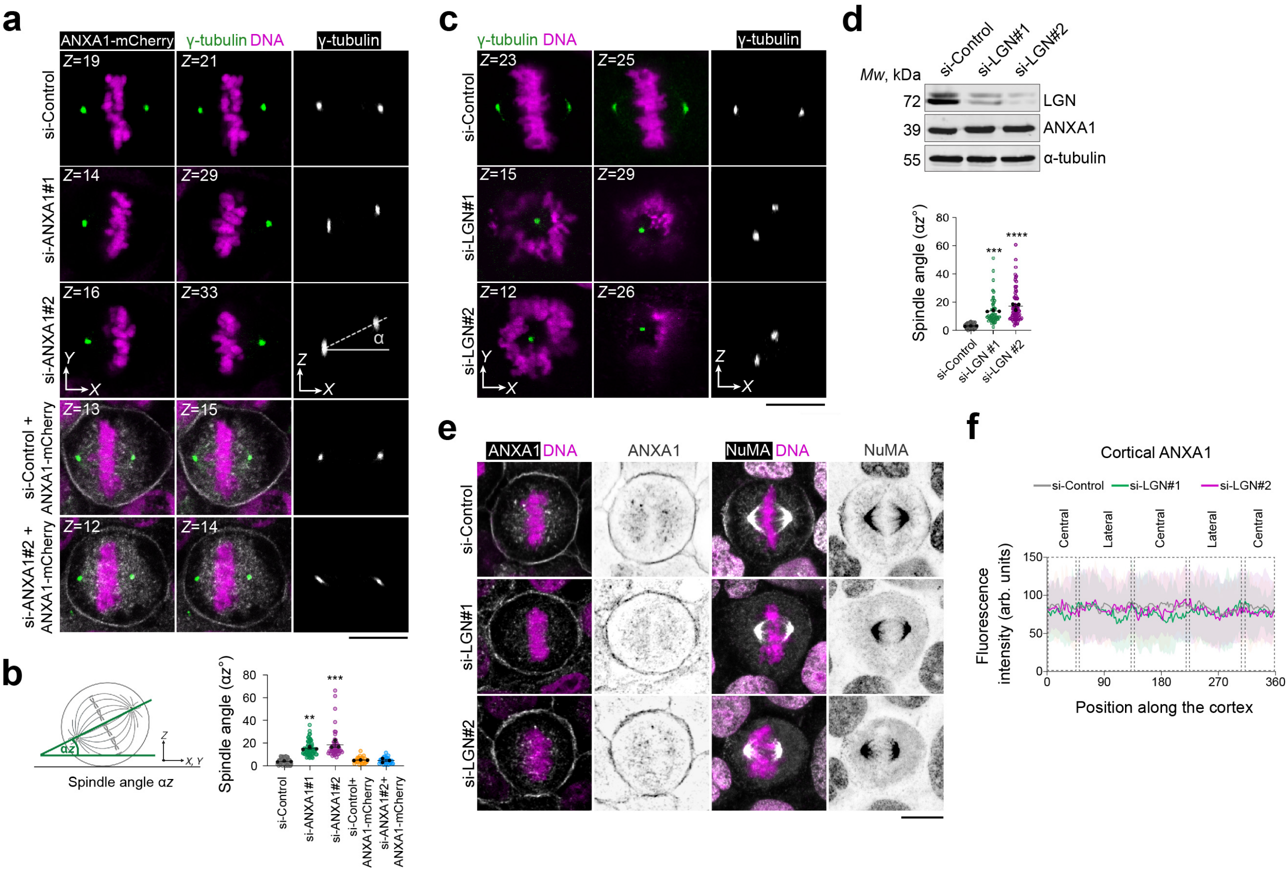

Supplementary Fig. 3 ANXA1 acts upstream of LGN to ensure proper mitotic spindle orientation. (a) Confocal images of representative MCF-10A cells transfected with si-Control, si-ANXA1#1 or si-ANXA1#2, expressing or not ANXA1-mCherry and stained for γ-tubulin (green), and counterstained with DAPI (DNA, magenta). *Z* projections in the *XY* view and *XZ* view are shown. Scale bars, 5 μm. **(b)** Mitotic spindle angles in siRNA-transfected cells expressing or not ANXA1-mCherry (si-Control: *n* = 71; si-ANXA1#1: *n* = 65; si-ANXA1#2: *n* = 49; si-Control + ANXA1-mCherry: *n* = 30; si-ANXA1#2 + ANXA1-mCherry: *n*= 30). One-way ANOVA with Tukey's test, ** *P* = 0.002; *** *P* = 0.001. **(c)** Confocal images of representative MCF-10A cells transfected with si-Control, si-LGN#1 or si-LGN#2 stained for γ-tubulin (green) and counterstained with DAPI (DNA, magenta). Z projections in the XY view and XZ view are shown. Scale bars, 5 μm. **(d)** Top: Western blotting of extracts from MCF-10A transfected with si-Control, si-LGN#1 or si-LGN#2. Bottom: Mitotic spindle angles in siRNA-transfected cells (si-Control: *n* = 68; si-LGN#1: *n* = 50; si-LGN#2: *n* = 67). One-way ANOVA with Tukey's test, *** *P* = 0.0006; **** *P* = 0.0001. **(e)** Confocal images of representative MCF-10A cells stained for ANXA1 or NuMA (grey) and counterstained with DAPI (DNA, magenta).

Scale bars, 10 μm. **(f)** Average cortical fluorescence intensity profiles of ANXA1 from metaphase siRNA-transfected cells (si-Control *n* = 41; si-LGN#1 *n* = 26; si-LGN#2 *n* = 25). All data are presented as mean ± s.e.m. from 3 independent experiments. arb. units (arbitrary units). Source data are provided as a Source Data file.

Supplementary Fig. 4

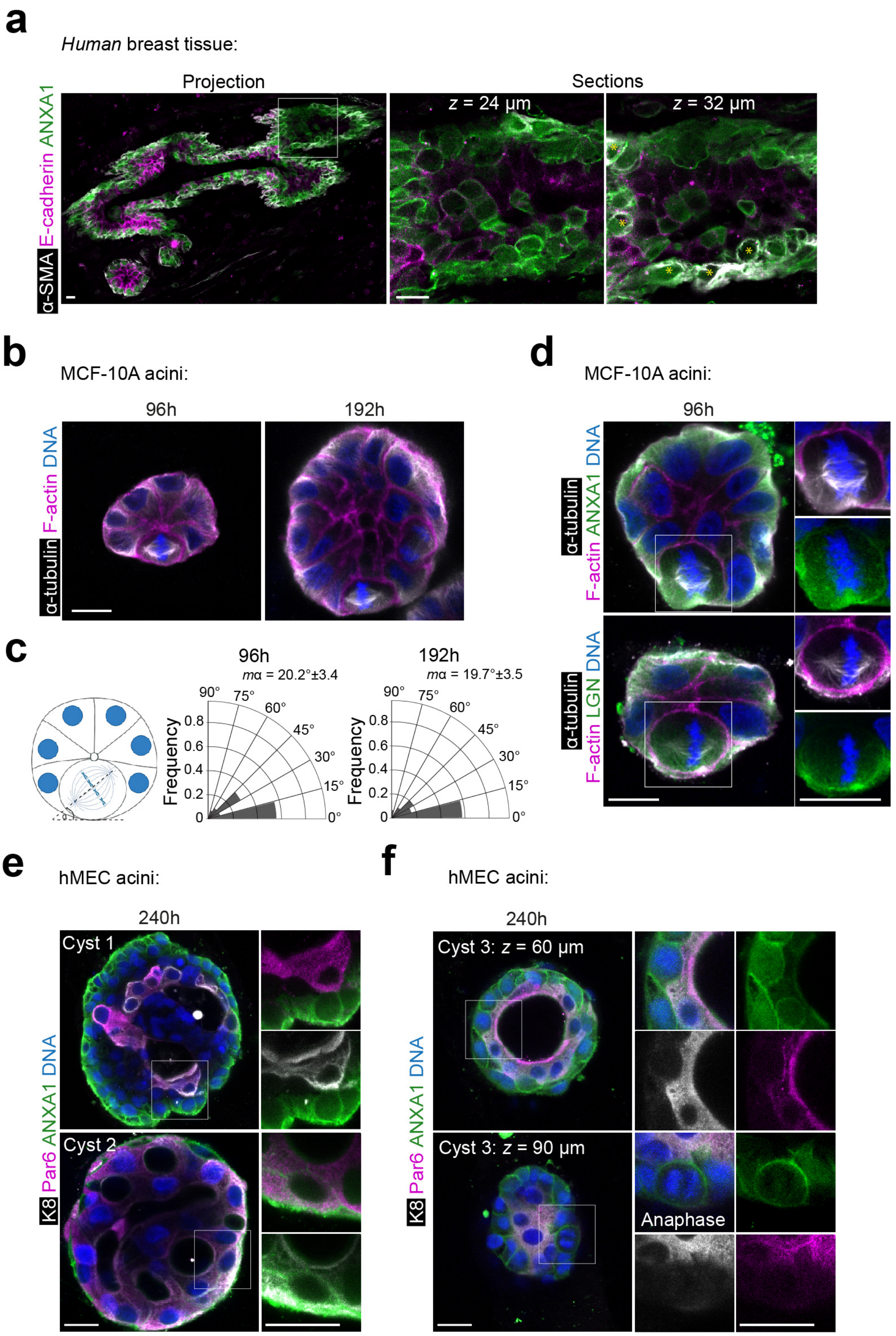

Supplementary Fig. 4 Characterisation of ANXA1 expression and localisation and OCDs in human mammary epithelial cells *in situ* and 3D culture. (a) Confocal images of representative

human healthy breast cryosections (50 µm-thick) stained for ANXA1 (green), α-SMA (grey) and E-cadherin (magenta). **(b)** Confocal images of representative MCF-10A acini stained for F-actin (magenta) and α-tubulin (grey) and counterstained with DAPI (DNA, blue). **(c)** Spindle angle frequencies and mean angles ($m\alpha$) (3 independent experiments, 96h: n = 30 acini; 192h: n = 30 acini). **(d)** Confocal images of representative MCF-10A acini stained for F-actin (magenta), α-tubulin (grey) and LGN or ANXA1 (green), and counterstained with DAPI (DNA, blue) (3 independent experiments). **(e)** Confocal images of representative hMEC acini forming multiple small lumens, stained for Par6 (magenta), K8 (grey) and ANXA1 (green), and counterstained with DAPI (DNA, blue) (2 independent experiments). **(f)** Confocal images of representative hMEC acini forming a single central lumen, stained for Par6 (magenta), K8 (grey) and ANXA1 (green), and counterstained with DAPI (DNA, blue) (2 independent experiments). Data are presented as mean ± s.e.m. All scale bars, 10 µm. Source data are provided as a Source Data file.

Fiji custom macro for quantification of cortical protein fluorescence intensities during prometaphase

```
macro elliptic_slicing {

//number of points to measure along the cell perimeter
k=180;
//length (in pixels) of the line for each measure:
px=15;
//starting position of the scan line relative to the ellipses long axis
S=90;

titre=getTitle();
getDimensions(x,y,nb_channel,o,t);
run("Set Measurements...", "area mean standard min bounding fit redirect=None decimal=3");
b=57.29578;
m=nResults;
IJ.deleteRows(0, m-1);
getPixelSize(unit, pw, ph, pd);
P=pd/pw;
run("Properties...", "unit=pixel pixel_width=1.0000 pixel_height=1.0000 voxel_depth="+(P)+"
origin=0,0");

            selectWindow(titre);
            run("Set... ", "zoom=150");
            setTool("Freehand");

//message prompting the user to set the ellipse:
            title = "cell center";
            msg = "use the Freehand tool to draw\n the cell's perimeter\n select the best z-
level\n then click \"OK\".";
            waitForUser(title, msg);

//measuring the ellipses center and size:
            run("Fit Ellipse");
            run ("Measure");
            x0=getResult("BX", (0));
            x1=getResult("Width", (0));
                        M=getResult("Major", (0));
                        m=getResult("Minor", (0));
                        a=getResult("Angle", (0));
                        E=sqrt(M*M-m*m)/M;
            y0=getResult("BY", (0));
            y1=getResult("Height", (0));
      //center of the ellipse:
            X0=x0+x1/2;
            Y0=y0+y1/2;

xx=newArray(k);
yy=newArray(k);

      for (i=0; i<k; i++) {
      xx[i]=(i);
      l=(360*i/k-a+S)/b;
      L=(360*i/k+S)/b;
      dia=sqrt(m*m/(1-E*E*cos(L)*cos(L)))/2;
```

```
            n1=dia-px/2;
            n2=dia+px/2;
        selectWindow(titre);
        makeLine(X0+n1*cos(l), Y0+n1*sin(l), X0+n2*cos(l), Y0+n2*sin(l));
                run ("Measure");
                yy[i]=getResult("Max", (0));
                IJ.deleteRows(0, 0);
        print(yy[i]);
        };
Plot.create("plot1", "X", "Y", xx, yy);
}
```

Representative examples of the gating strategies used to purify stable MCF-10A expressing GFP-LGN, EB3-GFP or ANXA1-mCherry

1. Purification of MCF-10A cells expressing GFP-LGN or EB3-GFP

Negative Control: Wild-type/non-transfected MCF-10A cells

Cell gating & exclusion of debris → Gating of GFP+ cells

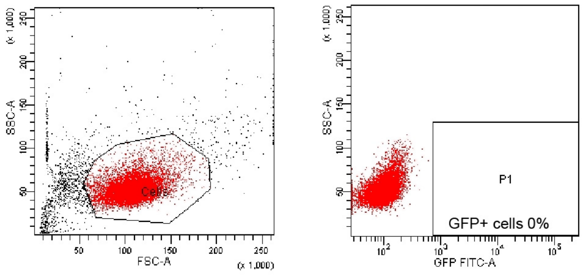

Tube: Non-transfected

Population	#Events	%Parent	%Total
All Events	10,000	####	100.0
Cells	8,550	85.5	85.5
P1	0	0.0	0.0

Positive Control: MCF-10A cells expressing pTK14-GFP

Cell gating & exclusion of debris → Gating of GFP+ cells

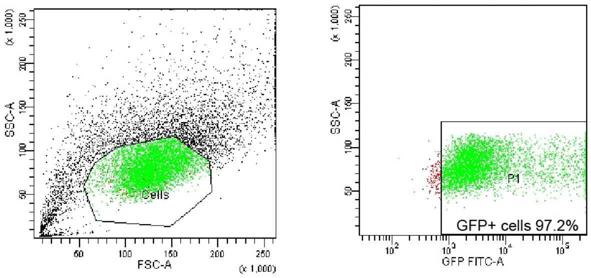

Tube: pTK14-GFP

Population	#Events	%Parent	%Total
All Events	10,000	####	100.0
Cells	5,939	59.4	59.4
P1	5,774	97.2	57.7

MCF-10A expressing pTK14-GFP-LGN

Cell gating & exclusion of debris → Gating of GFP+ cells

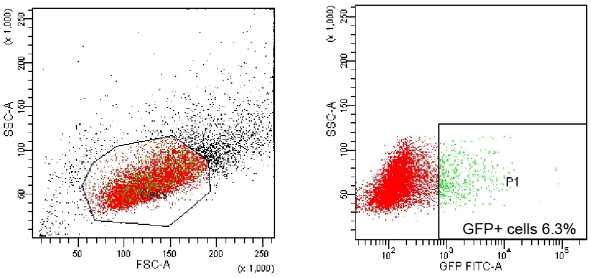

Tube: pTK14-GFP-LGN

Population	#Events	%Parent	%Total
All Events	10,000	####	100.0
Cells	7,895	79.0	79.0
P1	495	6.3	5.0

MCF-10A expressing pTK14-EB3-GFP

Cell gating & exclusion of debris → Gating of GFP+ cells

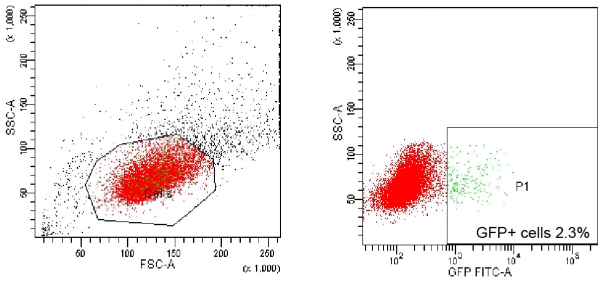

Tube: pTK14-EB3-GFP

Population	#Events	%Parent	%Total
All Events	10,000	####	100.0
Cells	8,715	87.2	87.2
P1	201	2.3	2.0

2. Purification of MCF-10A expressing ANXA1-mCherry

Negative Control: Wild-type/non-transfected MCF-10A cells

Cell gating & exclusion of debris → Gating of mCherry+ cells

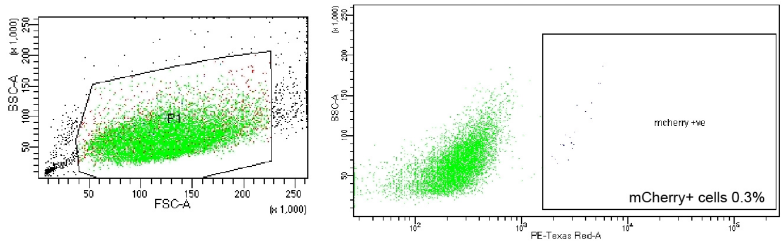

Tube: **Non-transfected**

Population	#Events	%Parent	%Total
All Events	10,000	####	100.0
P1	9,187	91.9	91.9
P2	7,915	86.2	79.1
mcherry +ve	20	0.3	0.2

Positive Control: MCF-10A cells expressing mCherry

Cell gating & exclusion of debris → Gating of mCherry+ cells

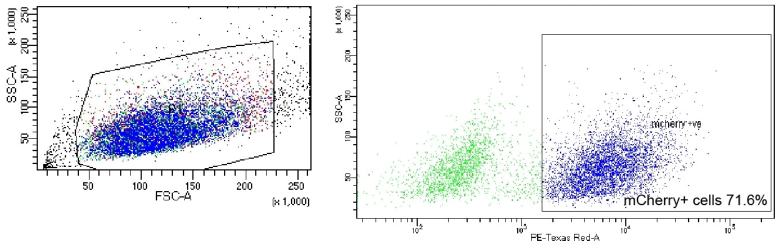

Tube: **pTK93-mCherry**

Population	#Events	%Parent	%Total
All Events	10,000	####	100.0
P1	9,307	93.1	93.1
P2	7,718	82.9	77.2
mcherry +ve	5,527	71.6	55.3

MCF-10A cells expressing ANXA1-mCherry

Cell gating & exclusion of debris → Gating of mCherry+ cells

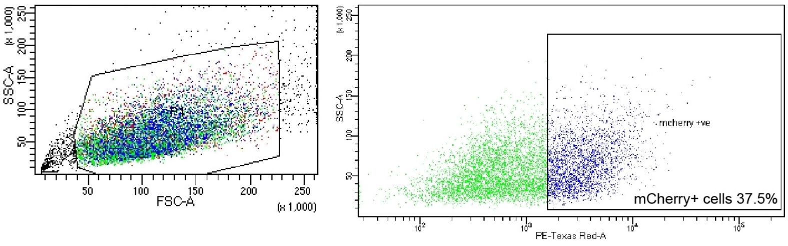

Tube: **pTK93-ANXA1-mCherry**

Population	#Events	%Parent	%Total
All Events	10,000	####	100.0
P1	9,084	90.8	90.8
P2	7,369	81.1	73.7
mcherry +ve	2,761	37.5	27.6
