# Supplementary material for: Annexin A1 is a polarity cue that directs mitotic spindle orientation during mammalian epithelial morphogenesis
Source: Nat Commun. 2023 Jan 11;14:151. doi: 10.1038/s41467-023-35881-x (PMC9834401; doi:10.1038/s41467-023-35881-x)
Supplement: Supplementary file 3 — Description of additional Supplementary File [file 41467_2023_35881_MOESM3_ESM.pdf]

<u>Description of Additional Supplementary Files</u>

File Name: Supplementary Dataset 1. Summary of all the mass spectrometry proteomic data (two independent experiments) obtained by purifying the GFP-LGN protein complex using a GFP-TRAP-based pulldown in metaphase MCF-10A cells.

File Name: Supplementary Dataset 2. Summary of proteins in the LGN subnetwork in metaphase GFP-LGN expressing MCF-10A cells (two independent experiments).

File Name: Supplementary Dataset 3. Samples ID numbers and breast tissue donor anonymised information.

File Name: Supplementary Movie 1
Description: LGN spatiotemporal distribution in control cells. Clonal MCF-10A cells stably expressing GFP-LGN were transfected with si-Control RNA for 72h and treated with Hoechst as described in the Methods. Images corresponding to 30 planes spaced by 0.6 µm through the cell volume were collected every 3 min as described in the Methods. Maximum intensity projections of GFP-LGN (green) and Hoechst (DNA, magenta) are shown through time. Time in min format and scale bar = 5 µm.

File Name: Supplementary Movie 2
Description: ANXA1 knockdown impairs the dynamics of LGN and results in its unilateral distribution at the cortex. Clonal MCF-10A cells stably expressing GFP-LGN were transfected with si-ANXA1#1 RNA for 72h and treated with Hoechst as described in the Methods. Images corresponding to 40 planes spaced by 0.6 µm through the cell volume were collected every 3 min as described in the Methods. Maximum intensity projections of GFP-LGN (green) and Hoechst (DNA, magenta) are shown through time. Time in min format and scale bar = 5 µm.

File Name: Supplementary Movie 3
Description: ANXA1 knockdown impairs the dynamics of LGN and results in its central distribution at the cortex. Clonal MCF-10A cells stably expressing GFP-LGN were transfected with si-ANXA1#1 RNA for 72h and treated with Hoechst as described in the Methods. Images corresponding to 40 planes spaced by 0.6 µm through the cell volume were collected every 3 min as described in the Methods. Maximum intensity projections of GFP-LGN (green) and Hoechst (DNA, magenta) are shown through time. Time in min format and scale bar = 5 µm.

File Name: Supplementary Movie 4
Description: ANXA1 knockdown impairs the dynamics of LGN and results in its circumferential distribution at the cortex. Clonal MCF-10A cells stably expressing GFP-LGN were transfected with si-ANXA1#1 RNA for 72h and treated with Hoechst as described in the Methods. Images corresponding to 40 planes spaced by 0.6 µm through the cell volume were collected every 3 min as described in the Methods. Maximum intensity projections of GFP-LGN (green) and Hoechst (DNA, magenta) are shown through time. Time in min format and scale bar = 5 µm.

File Name: Supplementary Movie 5
Description: Mitotic spindle and chromosome dynamics in control cells. MCF-10A cells were transfected with si-Control RNA for 72h and treated with SiR-tubulin and Hoechst as described in the Methods. Images corresponding to 30 planes spaced by 0.6 µm through the cell volume were collected every 2.5 min as described in the Methods. Maximum intensity

projections of SiR-tubulin (green) and Hoechst (DNA, magenta) are shown through time. Time in min format and scale bar = 5 µm.

File Name: Supplementary Movie 6
Description: ANXA1 knockdown impairs mitotic progression and results in chromosome bridges during anaphase and telophase. MCF-10A cells were transfected with si-ANXA1#1 RNA for 72h and treated with SiR-tubulin and Hoechst as described in the Methods. Images corresponding to 40 planes spaced by 0.6 µm through the cell volume were collected every 2.5 min as described in the Methods. Maximum intensity projections of SiR-tubulin (green) and Hoechst (DNA, magenta) are shown through time. Time in min format and scale bar = 5 µm.

File Name: Supplementary Movie 7
Description: ANXA1 knockdown impairs mitotic progression and results in misaligned small mitotic spindles. MCF-10A cells were transfected with si-ANXA1#1 RNA for 72h and treated with SiR-tubulin and Hoechst as described in the Methods. Images corresponding to 40 planes spaced by 0.6 µm through the cell volume were collected every 2.5 min as described in the Methods. Maximum intensity projections of SiR-tubulin (green) and Hoechst (DNA, magenta) are shown through time. Time in min format and scale bar = 5 µm.

File Name: Supplementary Movie 8
Description: ANXA1 knockdown impairs mitotic progression and results in misaligned multipolar mitotic spindles. MCF-10A cells were transfected with si-ANXA1#1 RNA for 72h and treated with SiR-tubulin and Hoechst as described in the Methods. Images corresponding to 40 planes spaced by 0.6 µm through the cell volume were collected every 2.5 min as described in the Methods. Maximum intensity projections of SiR-tubulin (green) and Hoechst (DNA, magenta) are shown through time. Time in min format and scale bar = 5 µm.
